# Inferring Master Painters' Esthetic Biases from the Statistics of Portraits

**DOI:** 10.3389/fnhum.2017.00094

**Published:** 2017-03-09

**Authors:** Hassan Aleem, Ivan Correa-Herran, Norberto M. Grzywacz

**Affiliations:** ^1^Interdisciplinary Program in Neuroscience, Georgetown University Washington, DC, USA; ^2^Department of Neuroscience, Georgetown University Washington, DC, USA; ^3^Facultad de Artes, Universidad Nacional de Colombia Bogotá, Colombia; ^4^Department of Physics, Georgetown University Washington, DC, USA; ^5^Graduate School of Arts and Sciences, Georgetown University Washington, DC, USA

**Keywords:** complexity, symmetry, balance, processing fluency theory, neuroaesthetic space, neuroaesthetics, image statistics, portrait paintings

## Abstract

The Processing Fluency Theory posits that the ease of sensory information processing in the brain facilitates esthetic pleasure. Accordingly, the theory would predict that master painters should display biases toward visual properties such as symmetry, balance, and moderate complexity. Have these biases been occurring and if so, have painters been optimizing these properties (fluency variables)? Here, we address these questions with statistics of portrait paintings from the Early Renaissance period. To do this, we first developed different computational measures for each of the aforementioned fluency variables. Then, we measured their statistics in 153 portraits from 26 master painters, in 27 photographs of people in three controlled poses, and in 38 quickly snapped photographs of individual persons. A statistical comparison between Early Renaissance portraits and quickly snapped photographs revealed that painters showed a bias toward balance, symmetry, and moderate complexity. However, a comparison between portraits and controlled-pose photographs showed that painters did not optimize each of these properties. Instead, different painters presented biases toward different, narrow ranges of fluency variables. Further analysis suggested that the painters' individuality stemmed in part from having to resolve the tension between complexity vs. symmetry and balance. We additionally found that constraints on the use of different painting materials by distinct painters modulated these fluency variables systematically. In conclusion, the Processing Fluency Theory of Esthetic Pleasure would need expansion if we were to apply it to the history of visual art since it cannot explain the lack of optimization of each fluency variables. To expand the theory, we propose the existence of a Neuroesthetic Space, which encompasses the possible values that each of the fluency variables can reach in any given art period. We discuss the neural mechanisms of this Space and propose that it has a distributed representation in the human brain. We further propose that different artists reside in different, small sub-regions of the Space. This Neuroesthetic-Space hypothesis raises the question of how painters and their paintings evolve across art periods.

## Introduction

An important recent theory in art cognition is the Processing Fluency Theory (PFT) of esthetic pleasure, which claims the ease with which the brain processes the perceptual properties of an object contributes to its esthetic value (Reber et al., 2004); specifically, the hedonic value increases as the fluency of processing rises (Winkielman et al., 2003). The PFT has been highly influential, even though its mechanisms are unclear (Albrecht and Carbon, 2014; Graf and Landwehr, 2015) and its scope has limitations (Leder et al., 2004; Locher et al., 2007; Leder, 2013; Chatterjee and Vartanian, 2014). The theory has found applications in the cognitive studies of clarity (Whittlesea et al., 1990), marketing (Lee and Labroo, 2004), recognition memory (Whittlesea, 1993), and judgments of truth (Begg et al., 1992; Reber and Schwarz, 1999). In this paper, we propose to extend the applications of the PFT to the study of some aspects of the history of visual art.

Of the many visual properties studied under the PFT, symmetry, balance, and complexity have received much attention. Numerous studies have shown that individuals prefer objects with greater symmetry (Palmer, 1991; Enquist and Arak, 1994; Humphrey, 1997; Jacobsen et al., 2006) and balance (Poore, 1903; Arnheim, 1954; Locher et al., 1996). The brain has mechanisms dedicated to deal with visual symmetry because of its biological importance, e.g., in the processing of faces (Wolfe and Friedman-Hill, 1992; Gangestad et al., 1994; Herbert and Humphrey, 1996; Rhodes et al., 1998). Therefore, the brain inherently processes symmetry with fluency. Similarly, the brain has special mechanisms for detecting balance, as its absence indicates the need of attention (Itti et al., 1998). The case for complexity is less straightforward but largely consistent with the PFT. Some studies indicate that the preference for complexity lies on an inverted “U” curve, with people liking moderate amounts of complexity (Berlyne, 1971; Aitken, 1974; Nicki and Moss, 1975; Saklofske, 1975; Imamoglu, 2000) while other studies have found linear relationships (Stamps, 2002; Nadal et al., 2010). In general, the brain likes complexity because it has a direct relationship with the amount of information in the input. Accordingly, certain circuitries in the visual pathways of the brain have evolved to deal with as much information as possible (Atick and Redlich, 1992; Bialek et al., 1993; Stemmler and Koch, 1999; Balboa and Grzywacz, 2000). This is especially relevant in an evolutionary context, with the brain's inclination to extract the maximum amount of intelligible information from a natural scene (Kaplan and Kaplan, 1989; Heerwagen and Orians, 1995). These circuitries function best with image statistics related to natural scenes, which are orderly (Field, 1987; Ruderman and Bialek, 1994; Balboa and Grzywacz, 2003). This connection with the natural world provides a possible explanation for the inverted “U” behavior. Too much complexity may be violating the natural order, hurting fluency processing. Therefore, the behavior of visual complexity in relation to the PFT is a reflection of the struggle between the brain's attempt at maximizing information while still maintaining comprehensibility.

In accordance with the principles of the PFT, we raise the hypothesis that visual artists, especially master painters will show biases toward optimizing fluency variables (i.e., those measuring visual properties like symmetry, balance, and complexity). Although the PFT is a theory for observers, visual artists also function as perceptual agents when performing their work (Bryson, 1983). They often struggle and revise their pieces until the right esthetic appeal emerges (Gombrich et al., 1977). Moreover, certain disturbances in perceptual abilities in artists are reflected in their artwork (Bogousslavsky, 2005; Rose, 2006; Chatterjee, 2015). However, even if the PFT were right, artists might not be able to optimize the various fluency variables independently. One reason is the complex relationship between the variables. For example, making a painting more symmetric may reduce its complexity. Another reason is that different painters may use different media, which may favor one variable over another. Hence, another hypothesis is that painters may be forced to make choices to optimize some variables at the cost of others.

To test these hypotheses, we first developed computational measures of symmetry, balance, and complexity. We then investigated the statistics of the relationship between these variables in both master paintings and control photographs to study the choices that artists make about these characteristics. Our control photographs consisted of both photos of individuals in controlled poses, as well as quickly taken photos with minimal artistic intent. Comparison of the latter with master paintings allowed us to test whether painters produced art with more symmetry, balance, and complexity than what one would get in spontaneous, quickly snapped photographs. On the other hand, comparison of master paintings with the ideally balanced and symmetric frontally posed controls allowed us to test whether artists optimized their work with respect to these fluency variables. Our investigation adds to previous work that has probed statistical properties of visual art (Graham and Redies, 2010). That work found, for example, interesting overlap in the power spectra of both faces in portrait paintings and natural scenes in photographs (Redies et al., 2007a). Here we concentrated on different statistical variables, which were more closely related to the PFT. The focus of the study reported here was on portrait paintings during the Early Renaissance period. We limited our studies to portraits because they were relatively simple (e.g., typically composed vertically). This simplicity aided in both avoiding contextual complexity and reducing the computational difficulty in the measurements. We chose to focus on the Early Renaissance since portraiture as an art form first emerged during this period (Pope-Hennessy, 1966).

## Materials and methods

### Images analyzed

#### General properties of the images

All images were digitized to a maximum resolution of 1,024 × 1,024 pixels due to computational cost. The minimum number of pixels in the horizontal dimension, i.e., the number of columns was 500. Such a minimum was necessary to allow enough precision in the estimation of the indices of symmetry, balance, and complexity along this dimension. If the number of columns was uneven, one column was detracted from the end of the image to allow precise computation of left-right symmetry and balance in the image. Finally, although the images were originally in color, we converted them into 8-bit grayscale through the colorimetric method (Poynton, 1998). This conversion was necessary because, in this paper, we focus on symmetry, balance, and complexity for the relative intensities of the images.

#### Portrait paintings

We analyzed 153 portrait paintings of 26 master painters from the Early Renaissance, which we defined as those born between 1370 and 1450. Portrait paintings were only included if they each contained one main individual as the subject. They were painted in oil, tempera, frescos, or a mixture of these materials. All paintings were obtained from the online database, “Artstor Digital Library” (library.artstor.org). If the painting in Artstor had a frame, we removed it before the statistical estimations, except if the painter had painted it.

A complete list of the paintings and controls in this paper appears in the Supplementary Materials, including the values of some of the most relevant measurements (using the same conventions as in **Figures 6**, **7**).

#### Posed controls

Six graduate students and three professors from Georgetown University were chosen as volunteers for these controls. Photographs of the volunteers were taken while sitting at frontal, 45°, and 90° poses. Thus, we obtained 27 posed-control photographs (three each of nine subjects). Our control posed photographs had the direction of the face, shoulders/chest, and eyes fully congruent, and always rotated around a vertical axis. These well-controlled conditions served to reduce statistical variability in the fluency variables in this group, allowing us to use a relatively small number of photographs. To reduce variability further, all the photographs were taken indoors with identical lighting conditions by using a Canon EOS 700D/Rebel T5i camera fitted with an EF-S 18-55 mm F3.5-5.6 IS STM lens (Canon USA, Inc., Melville, New York, USA).

Each volunteer signed a written consent based on an approved Institutional Review Board protocol.

To compare these controls with the master paintings more directly, we attempted to classify the poses in the latter. Such a classification in artistic paintings could only be approximate. Artists varied at least three different variables within the pose structure, including rotating the face, shoulders/chest, and the eyes separately. Furthermore, these three variables were occasionally modulated parametrically along both the vertical and the horizontal axes. Artists most likely exploited these modulations to increase complexity in their works (Pope-Hennessy, 1966). This large number of artistic degrees of freedom created a difficult problem for an objective, unambiguous classification of pose. Nevertheless, to get an approximation, each of the three authors performed a subjective classification in a single-blinded manner with *a priori* exclusion criteria. One of these criteria were the use of only the direction of the face and shoulders (i.e., excluding the eyes because of their small sizes). Furthermore, the criteria allowed rotation only around a vertical axis. Finally, to be classified, the directions the face and shoulders needed to be subjectively congruent. Otherwise, the painting was considered unclassified. The subjectively classified poses were limited to three categories to match the posed controls, namely, frontal, angled, and profile. After the three authors independently classified the paintings, we used only those that had unanimous classifications. This process resulted in 103 classified paintings. Of these, 69 were angled, 31 were profile, and 3 were frontal.

#### Quickly snapped photographs

We wanted the last set of controls to be images in which no deliberate artistic effort existed. Therefore, instead of using publicly available photographs that may contain artistic intent, we selected photographs from a collection of casual, quickly snapped, and as-unposed-as-possible photographs taken by the authors with their iPhone 6 (Apple, Cupertino, California, USA). Rejection criteria were similar to those of portrait paintings and posed controls, i.e., photographs had to contain one main, recognizable subject and not be more than 90° in pose. We also rejected photographs with any blurred portions. Two of the authors blindly selected appropriate photographs from a pool of 230 images. After consensual selection, only 38 survived the rejection criteria, thus having minimal posing, framing, and artistic effort. Each of the subjects signed a written consent to have his or her photograph used in this study based on an approved Institutional Review Board protocol.

All control photographs, posed or quickly snapped, are available upon request.

### Statistical measures

We developed computational measures of symmetry, balance, and complexity, and analyzed them statistically using MATLAB R2015a (MathWorks, Natick, Massachusetts, USA), using scripts developed specially for this project.

To make the reading of the following sections on the computational measures more accessible, we begin each text with a paragraph that provides the physical intuition of the proposed calculations. We hope that these paragraphs will allow the reader to understand the rationale even by skipping the equations. These, in turn, appear after the introductory paragraphs.

#### Symmetry and asymmetry indices

Symmetry in images has previously been defined in a variety of manners. The definitions include different types of symmetry, especially rotational, reflectional (along different axes), or translational. While many algorithms exist for these measurements, our method is similar in nature to the ones concerning vertical bi-lateral symmetry (O'Mara and Owens, 1996). In this paper, we only address vertical symmetry, since it is the predominant form in faces and portrait paintings. We specifically focus on symmetry across the center of the canvas, irrespective of the location of the face. To measure the index of symmetry, we subtract the intensities of each pixel and its pair across the midline. For identical intensities, the result is zero, indicating that those pixels are symmetric. If the intensities are not identical, the difference can range up to the maximum intensity in our image, namely, 255. To normalize these results, we take the root mean square of all of the subtractions and divide the result by this maximum. This normalization procedure results in an Index of Asymmetry that goes from 0 (no asymmetry) to 1 (complete asymmetry). The Index of Symmetry, therefore, is one minus the Index of Asymmetry.

Let I_k, j_ be the intensity of the pixel in Row k and Column j. Let the number of rows and columns be *N*_r_ and *N*_c_ respectively. The latter is even. Finally, let I^*^ be the maximally possible intensity. We build pair-of-pixels asymmetry measures as:

$$A_{k,\;j}=\frac{I_{k,\frac{N_{c}}{2}\;+\;1\;-\;j}-I_{k,\frac{N_{c}}{2}\;+\;j}}{I^{*}},\;1\leq j\leq \frac{N_{c}}{2}.$$

We define the overall (medial vertical) asymmetry index from this equation as:

(1) $$A=\sqrt{2\frac{\sum_{k\;=\;1}^{N_{r}}\sum_{j\;=\;1}^{N_{c}}A_{k,\;j}^{2}}{N_{r}N_{c}}}.$$

This asymmetry index is what appears in **Figures 4**, **6**, **7**. The corresponding (medial-vertical) symmetry index is 1 – A.

#### Balance and imbalance indices

The main idea behind pictorial balance is that features of the image have a spatial distribution that is as homogeneous as possible. Because portraits are vertical, one reasonable measurement of balance is along the horizontal dimension, which is what we do here. There are multiple definitions of pictorial balance, from which we consider three prominent ones. The first is suggested by the bodies of literature from Art, and History and Psychology of Art (Ross, 1907; Arnheim, 1954). This statistic measures artistic balance as commonly conceptualized in Physics, i.e., weighing masses with the distance to the fulcrum. Therefore, something further away from the fulcrum has a greater perceived weight. For our measurement, light intensities and the balance line are the equivalents of masses and the fulcrum respectively. Second, we consider a statistic that bypasses the distance weighing and simply compares the sum of the total mass (integrals) on the two sides of the balance line. This measure is similar to the comparing of intensities at the excitatory and inhibitory regions of an orientation-selective receptive field (Hubel and Wiesel, 1959; Hirsch and Martinez, 2006; Rapela et al., 2010). Such receptive fields basically perform the comparison by weighing one side with a +1 and the other with a −1. If the sides have similar content, then the receptive-field integral yields a zero response, indicating balance. Lastly, we consider a measure that compares mean intensities at the two sides of the balance line. This measure takes into account the spatial normalization common in some models of visual function (Heeger, 1992). We call these three measures the physicalist, integral, and mean models of pictorial balance respectively. Unlike the measurement for symmetry, these types of balance can be measured from different points other than the midline itself. We consider the simplest case, balance through the middle of the image, in **Figures 4–7**. In **Figure 2**, we explore other possible locations of balance lines as related to important features of the face.

Let the *x*_*b*_ be the location of the balance line (1 < *x*_*b*_ < *N*_*c*_). To define the integral balance, integrals of the intensities of each side of this line are taken:

(2) $$J_{L}^{\left(i\right)}\left(x_{b}\right)=\sum_{k\;=\;1}^{N_{r}}\sum_{j<x_{b}}I_{k,j},J_{R}^{\left(i\right)}\left(x_{b}\right)=\sum_{k\;=\;1}^{N_{r}}\sum_{j>\;x_{b}}I_{k,j},$$

where the superscript “(i)” indicates “integral,” and subscripts L and R mark left and right of the midline respectively. We define the integral-imbalance index as:

(3) $$B_{*}^{\left(i\right)}\left(x_{b}\right)=\{\begin{array}{cc} \frac{\left|J_{L}^{\left(i\right)}\left(x_{b}\right)\;-\;J_{R}^{\left(i\right)}\left(x_{b}\right)\right|}{J_{L}^{\left(i\right)}\left(x_{b}\right)\;+\;J_{R}^{\left(i\right)}\left(x_{b}\right)} & if\;J_{L}^{\left(i\right)}\left(x_{b}\right)\;+\;J_{R}^{\left(i\right)}\left(x_{b}\right)\;>0\;\\ 0 & otherwise \end{array},$$

and the integral-balance index as one minus this quantity. Thus, defined, both the integral-balance and imbalance indices are between 0 and 1. In **Figures 4–7**, we display the Integral-imbalance index by the medial vertical line, i.e.:

(4) $$B_{V}^{\left(i\right)}=\;B_{*}^{\left(i\right)}\left(\frac{N_{c}+1}{2}\right).$$

The physicalist-imbalance index is similar to Equations (2) and (3), except that it weighs the intensities with the distances to the balance line, i.e.:

$$\begin{array}{l} J_{L}^{\left(p\right)}\left(x_{b}\right)=\sum_{k\;=\;1}^{N_{r}}\sum_{j<\;x_{b}}|x_{b}-j|I_{k,\;j},\\ J_{R}^{\left(p\right)}\left(x_{b}\right)=\sum_{k\;=\;1}^{N_{r}}\sum_{j<\;x_{b}}|x_{b}-j|I_{k,\;j}, \end{array}$$

and

(5) $$B_{*}^{\left(p\right)}\left(x_{b}\right)=\{\begin{array}{cc} \frac{\left|J_{L}^{\left(p\right)}\left(x_{b}\right)\;-\;J_{R}^{\left(p\right)}\left(x_{b}\right)\right|}{J_{L}^{\left(p\right)}\left(x_{b}\right)\;+\;J_{R}^{\left(p\right)}\left(x_{b}\right)} & if\;J_{L}^{\left(p\right)}\left(x_{b}\right)\;+\;J_{R}^{\left(p\right)}\left(x_{b}\right)\;>0\\ 0 & otherwise \end{array},$$

where the superscript “(p)” indicates “physicalist.”

Similarly, the mean-imbalance index is as in Equations (2) and (3), except that it uses the mean instead of the integrals, i.e.:

$$\begin{array}{l} J_{L}^{\left(m\right)}\left(x_{b}\right)=\{\begin{array}{cc} \frac{\sum_{k\;=\;1}^{N_{r}}\sum_{j\;<\;x_{b}}I_{k,j}}{N_{r}\left(x_{b}-1\right)} & if\;x_{b}\;is\;integer\\ \frac{\sum_{k\;=\;1}^{N_{r}}\sum_{j\;<\;x_{b}}I_{k,j}}{N_{r}floor\left(x_{b}\right)} & otherwise \end{array},\\ J_{R}^{\left(m\right)}\left(x_{b}\right)=\frac{\sum_{k\;=\;1}^{N_{r}}\sum_{j\;>\;x_{b}}I_{k,j}}{N_{r}\left(N_{c}-floor\left(x_{b}\right)\right)}, \end{array}$$

where the function *floor* rounds numbers downwards, and:

(6) $$B_{*}^{\left(m\right)}\left(x_{b}\right)=\{\begin{array}{cc} \frac{\left|J_{L}^{\left(m\right)}\left(x_{b}\right)\;-\;J_{R}^{\left(m\right)}\left(x_{b}\right)\right|}{J_{L}^{\left(m\right)}\left(x_{b}\right)\;+\;J_{R}^{\left(m\right)}\left(x_{b}\right)} & if\;J_{L}^{\left(m\right)}\left(x_{b}\right)+J_{R}^{\left(m\right)}\left(x_{b}\right)\;>0\\ 0 & otherwise \end{array},$$

where the superscript “(m)” indicates “mean.”

As for the integral-balance index, the physicalist- and mean-balance indices are one minus the corresponding imbalance indices.

Finally, in order to determine the optimal balance lines, we seek positions that minimize the imbalance indices, i.e.:

(7) $$\begin{array}{l} \Lambda_{i}=argmin_{x_{b}}B_{*}^{\left(i\right)}\left(x_{b}\right),\;\;\Lambda_{p}=argmin_{x_{b}}B_{*}^{\left(p\right)}\left(x_{b}\right),\\ \Lambda_{m}=argmin_{x_{b}}B_{*}^{\left(m\right)}\left(x_{b}\right). \end{array}$$

In this paper, we looked for the columns achieving these minima. Both Λ_*i*_ and Λ_*p*_ emerged from unique minima, but on occasion, we observed two local minima in the estimation of Λ_*m*_. In those cases, we selected the minimum with the lowest value of imbalance. Other selections were possible for Λ_*m*_, but our selection was good enough to capture the uncertainty inherent in the mean-balance index.

#### Quality- and thickness-of-balance indices

The balance and imbalance indices quantify how similar the two sides of a balance line are overall. However, similarity may be achieved by compensating a feature on, say, the top right of an image, with something in the bottom left. If so, although the image may have left-right balance overall, the balance may be imperfect at different heights of the image. We wanted to quantify how careful master painters were about this fine balance. For this purpose, we first determined the best balance position row by row in the painting. We then measured the error in these positions relative to the horizontal size of the image.

For the sake of brevity, we only show here how the calculation is performed for the integral balance since the methods are identical for the other forms of balance. We begin by adapting (Equations 2, 3, and 7) to be row by row, getting:

$$\begin{array}{l} J_{L}^{\left(i\right)}\left(x_{b,}\;k\right)=\sum_{j\;<\;x_{b}}I_{k,j},\;J_{R}^{\left(j\right)}\left(x_{b,}k\right)=\sum_{j\;>\;x_{b}}I_{k,j},\\ B_{*}^{\left(i\right)}\left(x_{b,}\;k\right)=\{\begin{array}{cc} \frac{\left|J_{L}^{\left(i\right)}\left(x_{b,}\;k\right)\;-\;J_{R}^{\left(i\right)}\left(x_{b,}\;k\right)\right|}{J_{L}^{\left(i\right)}\left(x_{b,}\;k\right)\;+\;J_{R}^{\left(i\right)}\left(x_{b,}\;k\right)} & \begin{array}{c} if\;J_{L}^{\left(i\right)}\left(x_{b,}\;k\right)\;+\;J_{R}^{\left(i\right)}\left(x_{b,}\;k\right)\\ \;>0 \end{array}\\ 0 & otherwise \end{array}, \end{array}$$

and

(8) $$\Lambda_{i}\left(k\right)=argmin_{x_{b}}B_{*}^{\left(i\right)}\left(x_{b,\;}k\right)\;.$$

This function is illustrated for different images in **Figure 3**. From this function, we estimate the Thickness-of-balance index as:

(9) $$\Delta_{i}=\frac{MAD_{k}\left(\Lambda_{i}\left(k\right)\right)}{N_{c}},$$

where *MAD*_*k*_ is the median absolute deviation function with respect to *k*. As before, this index is a number from 0 to 1, representing the thickness of the function Λ_*i*_ relative to the canvas. Thus, we can define the Quality-of-balance Index as one minus the result expressed in Equation (9).

#### Complexity of order 1

**Figure 1:**
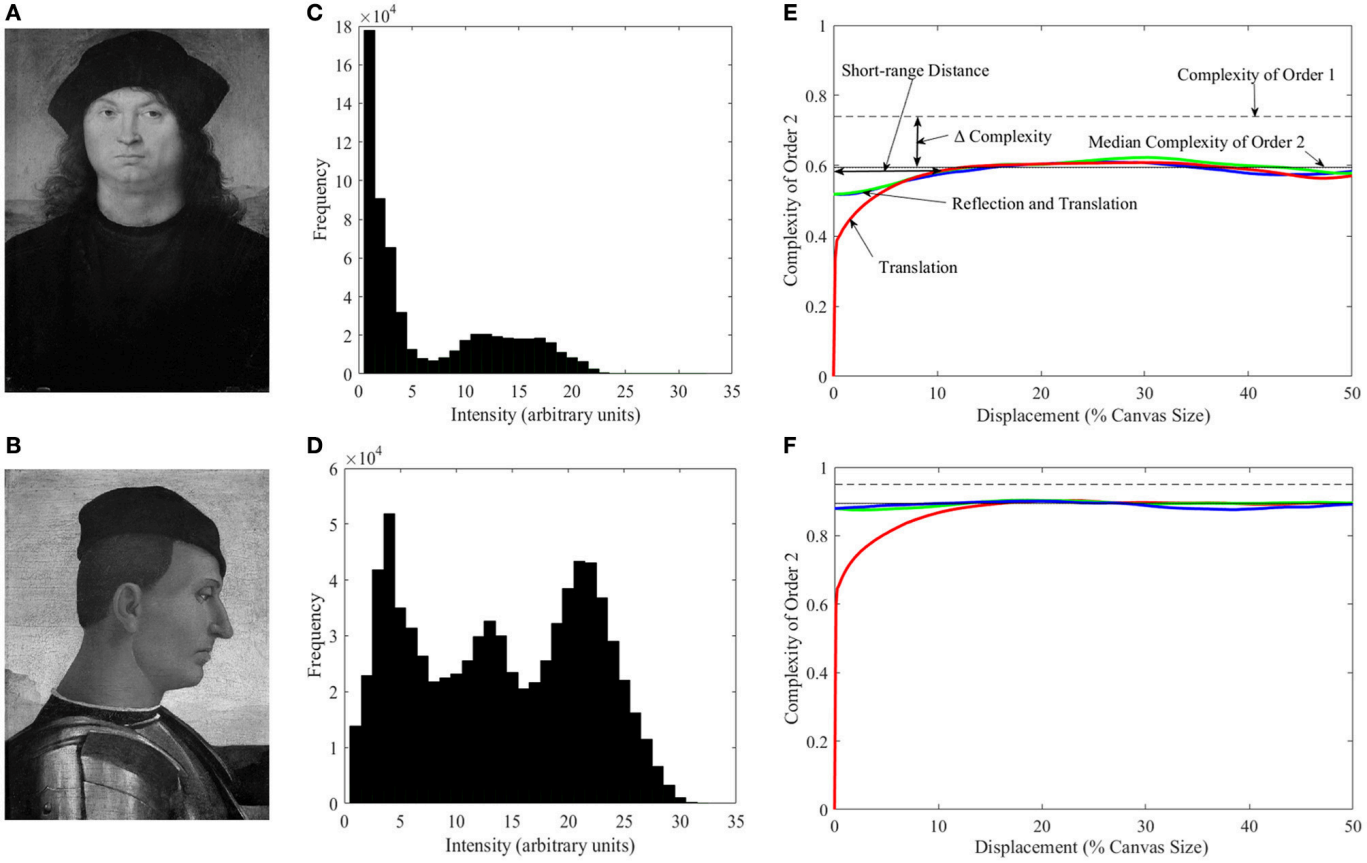
**Illustration of different types of complexity**. **(A)** *Portrait of a Man* by Pietro Perugino^1^ (Galleria Borghese, Rome, Italy; Photo © 2006, Scala, Florence, Italy; reproduced by courtesy of the Ministero Beni e Att. Culturali, Rome, Italy). **(B)** *Camillo Vitelli* by Luca Signorelli (Berenson Collection, Villa I Tatti, Florence, Italy, reproduced by permission of © President and Fellows of Harvard College). **(C,D)** Histograms of the intensities in **A** and **B** respectively. **(E,F)** Plots of complexity as a function of the amount of translation (as percentage of the canvas size) in different isometric transformations for the Perugino and Signorelli paintings respectively. Complexity of Order 1 is marked with the dashed line. Complexities of Order 2 for left and right translations are represented by the red line (both directions give identical results). Blue and green lines present the linear combinations of horizontal reflection and translation to the left and right respectively. The solid line shows the median of Complexities of Order 2 across all possible transformations. The figure also illustrates the Short-range Distance, i.e., the range of distances for which Complexity of Order 2 varies with displacement. We define this distance as the threshold at which the red line crosses the median Complexity of Order 2 minus its median absolute deviation. “Δ Complexity” is defined as the difference between Complexity of Order 1 and the median Complexity of Order 2 within the Short-range Distance. We call this latter quantity the Short-range Complexity. The figure shows that the Perugino painting has lower Complexities of Order 1 and Order 2, and larger Δ Complexity than the Signorelli piece (see text for more details).

This measure looks at the overall distribution of intensities in a given image, with a greater spread in the distribution equating to greater complexity of order 1. Therefore, a very complex image would have a wide range of intensities such as white noise. In contrast, the simplest image would be a blank canvas with only one type of intensity. Figure 1 illustrates this measurement of complexity in the context of art. Figure 1A is the *Portrait of a Man* by Pietro Perugino and Figure 1C is the histogram of its intensities. The histogram has a large peak close to zero, reflecting the large portions of the canvas that are black. Thus, if someone asks, “What is the intensity of this random pixel?” the best guess is “zero,” because it will be often correct. This ease in answering this question contrasts with what Figures 1B,D show. This painting, *Camillo Vitelli* by Luca Signorelli has a distribution of intensities that are much more spread, making the probability of answering correctly much harder. The painting has much more richness of intensities. Consequently, we should say that it is more complex than the painting in Figure 1A. To quantify Complexity of Order 1, we use the analog of entropy (i.e., the degree of disorder). In this paper, we do so by using Shannon's definition of informational complexity (Shannon and Weaver, 1949). For discussions regarding methods of measuring visual complexity using information theory and other definitions see (Forsythe et al., 2008; Graham and Redies, 2010; Nadal et al., 2010).

Let pixels have intensities [0, …, I^*^] (from 0 to 255 in our collection). For Image Q, let intensity l have M_l_ occurrences in histograms such as those in Figures 1C,D. Then, we define the probability of Intensity l in Image Q as:

$$P_{Q}^{\left(1\right)}\left(l\right)=\frac{M_{l}^{\left(Q\right)}}{\sum_{j\;=\;0}^{I^{*}}M_{j}^{\left(Q\right)}}\;.$$

From this equation, we define Entropy of Order 1 for Image Q as:

$$H_{1}(Q)=-\sum_{l\;=\;0}^{I^{*}}P_{Q}^{\left(1\right)}(l)log_{2}\left(P_{Q}^{\left(1\right)}(l)\right)\;.$$

In practice, if $P_{Q}^{\left(1\right)}\left(l\right)=0$ for some l, the term is not included in the sum, avoiding the singularity of the logarithm. This is possible, because lim_x → 0_xlog(x) = 0.

To create an index of complexity out of this entropy, we divide it by its largest possible value given any arbitrary image. This largest value comes from large images for which every pixel has an intensity randomly picked from all possible values. Thus, $P_{Q}^{\left(1\right)}\left(l\right)=1/\left(I^{*}+1\right)$. Therefore, the maximal possible Entropy of Order 1 is:

(10) $$H_{max,\;1}=-\sum_{l\;=\;0}^{I^{*}}\frac{1}{I^{*}+1}log_{2}\left(\frac{1}{I^{*}+1}\right)=\;log_{2}(I^{*}+1).$$

Dividing *H*_1_(Q) by *H*_*max*,1_, one gets the Complexity of Order 1:

(11) $$C_{1}(Q)=-\sum_{l\;=\;0}^{I^{*}}P_{Q}^{\left(1\right)}(l)log_{I^{*}\;+\;1}(P_{Q}^{\left(1\right)}(l))\;.$$

By the definition of *H*_*max*, 1_, we have 0 ≤ C_1_(Q) ≤ 1, with 0 happening for single-tone images (i.e., the simplest ones) and 1 happening for images whose intensities are spread homogeneously through all possible values.

#### Complexity of order 2

A limitation of Complexity of Order 1 is that it does not capture the change in complexity due to spatial organization. If one scrambles the positions of the pixels in a painting, it looks more complex. However, because the distribution of intensities remains the same upon scrambling, the Complexity of Order 1 does not change. To capture the observed change of complexity, one must ask, “Given the intensity of a pixel, can one predict the intensity in another?” For nearby pixels, the answer is typically yes, because they often represent portions of the same surface (Field, 1987; Ruderman and Bialek, 1994; Balboa and Grzywacz, 2003). But the answer could also be yes for distant pixels if, e.g., the image was symmetric or periodic. To answer this question generally, one must perform arbitrary isometric (i.e., distance preserving) transformations of the image and ask if two juxtaposed pixels predict the intensities of each other. These transformations are generally linear combinations of translation, rotation, and reflection. However, here, we only include horizontal-translation and horizontal-reflection transformations, because we are dealing with vertical portraits. We then use entropy to quantify complexity, but this time using conditional probabilities of the intensity of a pixel predicting the intensity of another after the transformation. Because we use two pixels, we call the emerging quantity Complexity of Order 2. It has many interesting properties illustrated in Figures 1E,F. The colored lines in these panels illustrate Complexity of Order 2 for the Perugino and Signorelli paintings for different transformations, parametric on the amount of translation. One of the properties of Complexity of Order 2 that we will demonstrate mathematically is that it is always less or equal than that of Order 1. In addition, as predicted above, Complexity of Order 2 is low for short translations, rising for longer ones. The panels show that the median Complexity of Order 2 is lower for the Perugino painting (because of its larger darker areas). They also illustrate the range of distances for which Complexity of Order 2 varies with displacement (the translation required for the colored lines to reach the uncertainty of the median complexity). The fall in complexity from Order 1 to the median in the short range (Short-range Complexity) is larger for the Perugino painting because of its symmetry.

To define Entropy of Order 2 for Image Q and Isometric Transformation T, we begin by letting $M_{l_{1},\;l_{2}}^{\left(Q,\;T\right)}$ be the number of times a pixel with Intensity l_1_ is juxtaposed with a pixel with Intensity l_2_ after the transformation. From this number, we define the following conditional probabilities:

$$P_{Q}^{\left(2\right)}\left(l_{2}|l_{1},T\right)=\frac{M_{l_{1},\;l_{2}}^{\left(Q,\;T\right)}}{\sum_{k\;=\;0}^{I^{*}}\sum_{j\;=\;0}^{I^{*}}M_{k,\;j}^{\left(Q,\;T\right)}}.$$

From this definition, we define Entropy of Order 2 for Image Q and Transformation T as:

$$H_{2}\left(Q,T\right)=\text{​}-\sum_{l_{1}\;=\;0}^{I^{*}}P_{Q}^{\left(1\right)}\left(l_{1}\right)\sum_{l_{2}\;=\;0}^{I^{*}}P_{Q}^{\left(2\right)}\left(l_{2}|l_{1},T\right)log_{2}\left(P_{Q}^{\left(2\right)}\left(l_{2}|l_{1},T\right)\right).$$

The maximal value of Entropy of Order 2 occurs when the pixels are independent of each other. Thus, $P_{Q}^{\left(2\right)}\left(l_{2}|l_{1},T\right)=P_{Q}^{\left(1\right)}\left(l_{2}\right)$, which makes *H*_2_*(Q,T)* = *H*_1_*(Q)*. This equality has two consequences of interest: First, Entropy of Order 2 is smaller than Entropy of Order 1. Second, the maximal Entropy of Order 2 is the same as that from Order 1, namely, *H*_*max*, 1_ (Equation 10). A corollary of these two consequences is that Complexity of Order 2, i.e.:

(12) $$C_{2}\left(Q,T\right)=\frac{H_{2}\left(Q,T\right)}{H_{max,1}}\;.$$

is always smaller than Complexity of Order 1, i.e.:

(13) $$C_{2}\left(Q,T\right)\leq C_{1}\left(Q\right)\;.$$

#### Important face features

We wished to determine the balance definition (integral, physicalist, or mean) best capturing what artists used for their portraits. For this purpose, we compared the optimal balance lines (Equation 7) to important features in the face. We wrote a special MATLAB function to perform this comparison. This function first determined the positions of the primary eye (the eye closer to the center of the canvas, Tyler, 1998), secondary eye, and tip of the nose. The function did so by allowing the authors to click on these facial features and recording the pixel locations. We then calculated the horizontal distances between each of these facial features and the optimal balance lines. Distances were also calculated to the median position of the facial features and the center of the canvas. The balance line yielding the smallest distances to the facial features gave a clue to the strategy that artists used to balance their portraits.

## Results

### How master painters balance their portraits

#### Best definition of balance

**Figure 2:**
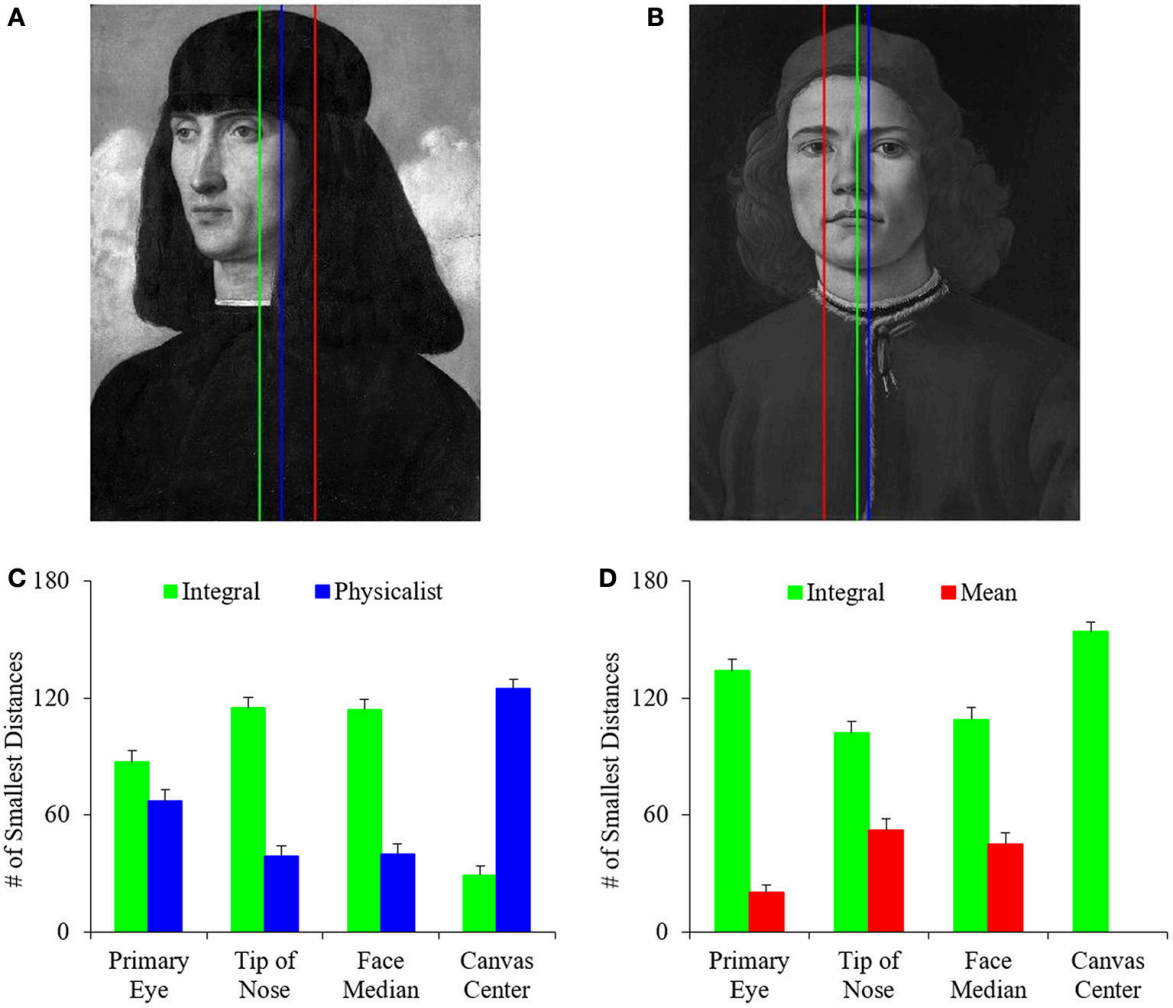
**Comparison of the different definitions of balance**. **(A,B)** Examples of where each of the best balance lines fall (Equation 7) for *Portrait of a Man* by Giovanni Bellini (Musée du Louver, Paris, France; reproduced by permission of © Reunion des Musees Nationaux, Paris, France) and *Portrait of a Young Man* by Sandro Botticelli (reprinted here with permission by the © National Gallery, London, Great Britain) respectively. These are Integral (green), Physicalist (blue), and Mean (red) balance lines. The integral balance line is closest to the center of the face in these two examples. **(C)** Number of times that Integral or Physicalist balance line was closest to the indicated feature of interest in 153 paintings. **(D)** Number of times that Integral or Mean balance line was closest to the indicated feature of interest. The Integral Balance line was significantly closer to important facial features, whereas the physicalist line was significantly closer to the center of the canvas.

In this study, we developed computational measures of symmetry, balance, and complexity, and investigated the relationship between these variables to probe the choices that artists of the Early Renaissance made about these quantities when painting portraits. Of these visual properties, the understanding of balance has perhaps been the most highly debated. Based on this debate, we have identified three different definitions of balance. They are the integral balance, physicalist balance, and mean balance (Equations 3, 5, and 6 respectively). Consequently, we began this study by determining which of these definitions matched best what we observed in portrait paintings. To do this, we first determined the positions in paintings in which each definition resulted in the greatest amount of vertical balance, or least imbalance (Equation 7). Not surprisingly, we found statistically significant positive correlations between these positions according to the different definitions of balance. For example, the Pearson linear correlation coefficients were 0.96 and 0.94 when plotting the best position of integral balance against those for the physicalist and mean balances respectively. We observed similar correlations for posed controls and quickly snapped photographs. These correlations make intuitive sense. They arise from the mathematical overlap of the definitions. However, a correlation between two definitions of balance does not mean that one of them is not better when inspected in detail. An illustration of the best positions obtained with the different definitions of balance can be seen in Figures 2A,B. Next, we compared these positions with important features in the paintings. These features were the center of the canvas, the primary eye, the tip of the nose, and the median of nose and eyes positions. Our aim was to determine which of the definitions yielded balance lines nearest to the salient features of the face. This definition would be deemed the best measure for balance. In the examples of Figures 2A,B, the integral-balance lines were closer to the tip of the nose or the middle of the face than the other lines. Was this advantage of the integral-balance line true for the majority of paintings? The answer can be seen in Figures 2C,D.

As suggested by Figures 2A,B, the different types of balance were not equal in their ability to approximate the positions of important facial features, with the advantage going to the integral lines. We reached this conclusion by calculating the number of times in 153 paintings that a specific balance line was closest to the facial features of interest. Because we had three candidate definitions of balance, under the null hypothesis that they were all equally good, we expected each candidate line to win about 153/3 = 51 times. This hypothesis set up a 3 × 2 χ^2^ test. With it, we rejected the hypothesis statistically for the tip of the nose (*p* < 0.03), primary eye (*p* < 0.0002), and the median of facial features (*p* < 0.04). Hence, the candidate balance lines do not approximate the work of artists equally well. We were then justified to make pairwise statistical comparisons between the different balance lines. As these comparisons showed, the integral balance line was significantly closer to the facial features of interest than the physicalist and mean lines. Statistical χ^2^ tests confirmed this conclusion for every spatial feature, except for the integral-physicalist comparison relative to the primary eye. For that comparison, the integral balance line was also better (Figure 2C), but the result was not statistically significant. (However, the physicalist balance line was always the closest to the canvas center. That was easy to explain, since if a candidate physicalist balance line is off-center in a particular direction, the larger distance weights in the opposite direction pull the line thus, bringing it back toward the middle of the canvas.)

Therefore, we conclude that the integral definition of balance captures what artist do better than the physicalist and mean ones. Hence, all of our subsequent analyses use the integral definition of balance.

#### Quality of integral balance lines

**Figure 3:**
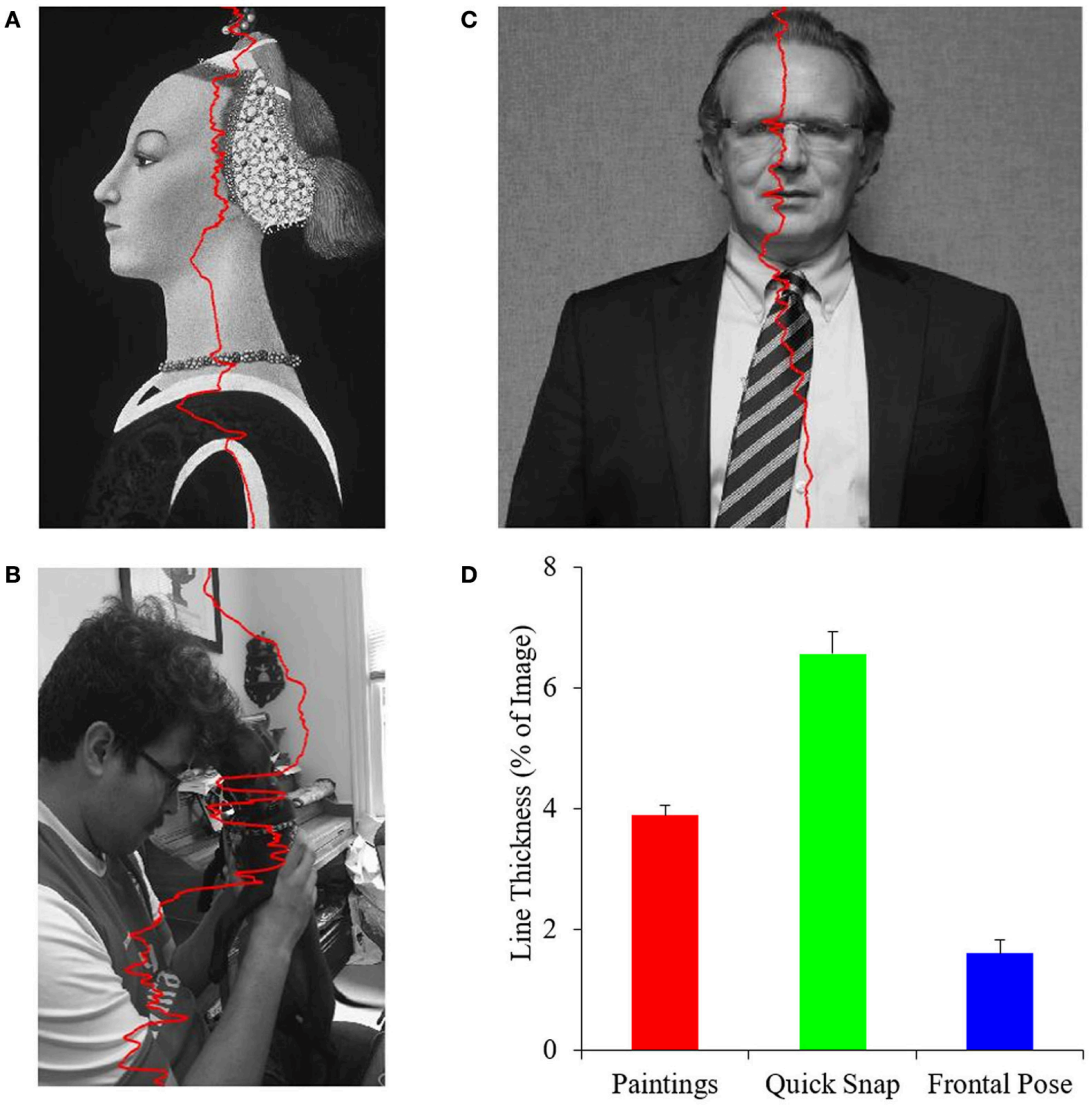
**The precision of integral balance in paintings**. **(A–C)** Examples of row-by-row balance lines (Equation 8) in *Portrait of a Lady* by Paolo Uccello (reprinted here thanks to the Open Access for Scholarly Content designation by the © Metropolitan Museum of Art, New York, United States), a quickly snapped photograph, and a frontally posed control respectively. The superimposed red curves are the balance lines. **(D)** Mean and standard error of balance-line thicknesses (Equation 9) for the different categories of images. Portrait artists of the Early Renaissance balanced paintings to a greater precision than quickly snapped photographs but not as well as in frontally posed controls.

A prediction of the PFT is that master painters would show biases toward optimizing balance in their portrait paintings. However, even if the PFT were right, artists might not optimize balance independently of other fluency variables. Our next goal was to get an understanding of exactly how well artists were balancing their paintings. We used two procedures to achieve this goal. In the one covered in the next section, we measured the imbalance index for all paintings. Here, we wanted to determine the precision of balance at different vertical heights of the image. To carry out this determination, we calculated the optimal position of balance as previously indicated, however in a row-by-row analysis. The result is a line of balance points (Equation 8) for each row as seen in Figure 3.

Figure 3 shows that master painters of the Early Renaissance made an effort to balance their portraits better than if just capturing a spontaneous image of their subjects. We appreciate this point by comparing the thicknesses of the balance lines in Figures 3A,B. The painting line shows more variation than that in the quickly snapped photograph. Thus, in Figure 3B, which shows a young man with his dog, the balance was pulled to the right at the top because of the bright wall. In turn, at the bottom, the white sleeve pulled the line to the left. By contrast, the balance line of the portrait painting showed less variation (Figure 3A).

However, Early Renaissance artists might not have balanced their paintings as well as they could. The balance points of the frontally posed photograph had much less variation (Figure 3C). Because the portrait painting was not frontal, controlling the position of balance was difficult. For example, the bright chin pulled the balance line to the left more than at other positions, increasing the variation of the balance positions.

A quantitative analysis of 153 portrait paintings, 38 quickly snapped photographs, and 9 frontally posed images confirmed that artists worked to balance their paintings but not to perfection. In this analysis, we compared the precision of balance in the three different categories of images. For each image, we calculated the line thickness (Equation 9). The analysis showed that portrait paintings yielded a lower line thickness than the quickly snapped photographs (Figure 3D). This difference in thickness was highly statistically significant (two-sided *t*-test, *t* = 5.4, 190 degrees of freedom, *p* < 2 × 10^−7^). However, portrait paintings yielded a larger line thickness than the frontally posed controls (Figure 3D – two-sided *t*-test, *t* = 3.4, 161 degrees of freedom, *p* < 0.0008).

### Quantification of the balance, symmetry, and complexity indices

**Figure 4:**
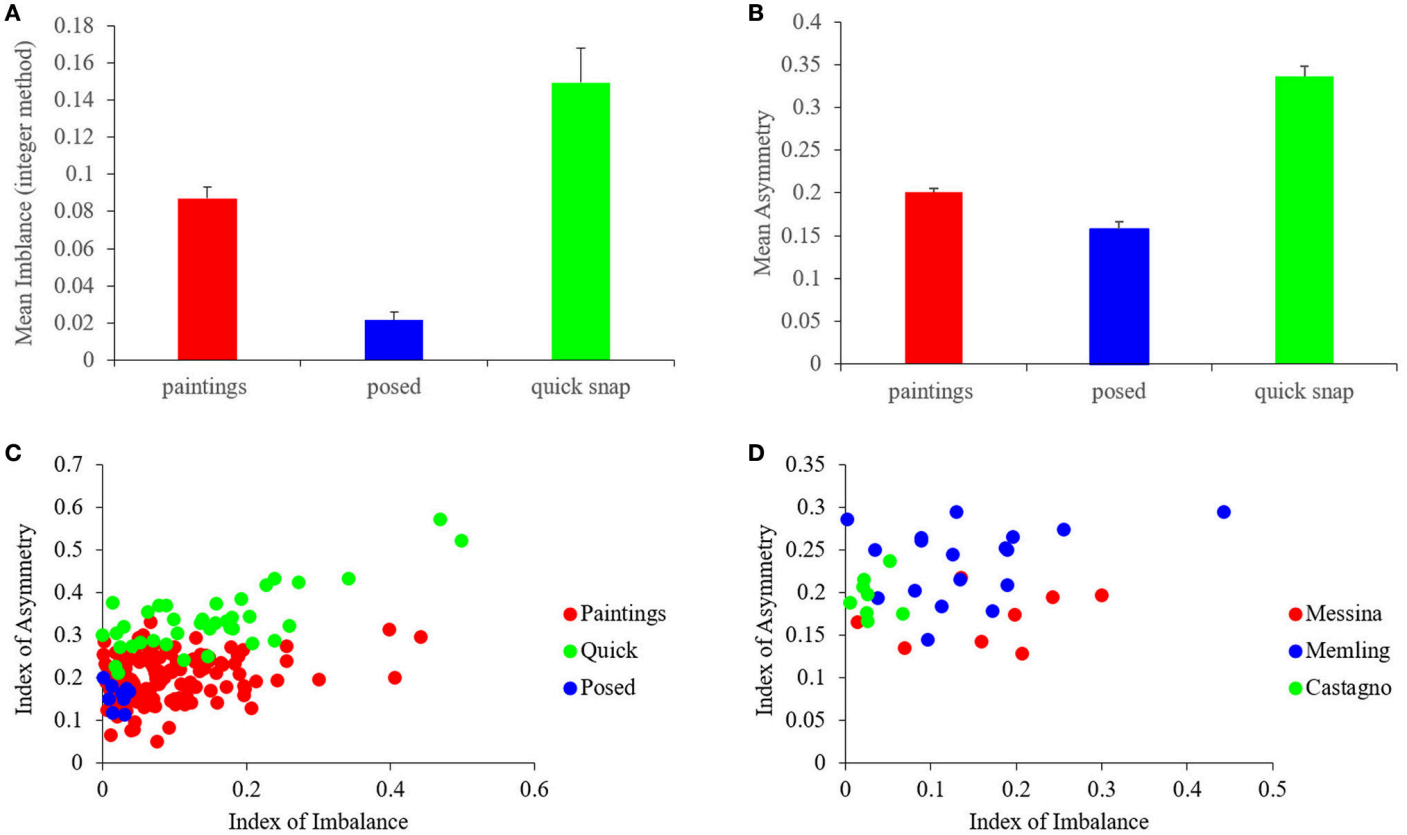
**Quantification of the Asymmetry and imbalance Indices**. **(A,B)** Mean and standard error of the Indices of Imbalance (Equation 4) and Asymmetry (Equation 1) respectively for the different categories of images. Artists balanced and symmetrized paintings more than quickly snapped photographs, but not as well as in frontally posed controls. **(C)** Scatterplots of the Indices of Asymmetry vs. the Indices of Imbalance for 153 paintings, 38 quickly snapped photographs, and 9 frontally posed. Both paintings and quickly snapped photographs exhibited positive correlations, while posed controls did not. **(D)** Same plot as in C but for three example artists, namely, Antonello da Messina, Hans Memling, and Andrea del Castagno. The indices for these artists cluster in different small sub-regions of the scatterplot.

Do master painters also show biases toward high symmetry and complexity, but not optimize them independently of the other fluency variables? To answer this question, we quantified symmetry and complexity. In addition, we studied the relationship of these fluency variables with balance. Here, we begin with the quantification of the Indices of Asymmetry (Equation 1) and Imbalance (Equation 4) for all the images in our study (Figure 4).

Figure 4 shows that the master painters also made an effort to symmetrize (balance) their portraits, but did not optimize symmetry (balance) independently of the other fluency variables. The result with the Imbalance Index (Figure 4A) was similar to that of precision-of-balance measures, confirming the conclusions derived from them (Figure 3D). The conclusions were then extended to the Asymmetry Index (Figure 4B). We confirmed this similarity with four statistical comparisons (two-sided Mann-Whitney tests), all showing significant differences. Two of the comparisons were for imbalance: Portraits-Quick (*p* < 0.001) and Portraits-Posed (*p* < 0.0007). The other two were for asymmetry: Portraits-Quick (*p* < 5 × 10^−18^) and Portraits-Posed (*p* < 0.01).

One reason for painters not to optimize balance and symmetry independently was that these variables showed a degree of correlation. A weak positive correlation between the Indices of Asymmetry and Imbalance was apparent for both portrait paintings and quickly snapped photographs in Figure 4C. Performing a Spearman's rank-order test, we confirmed that this correlation was statistically significant. The correlation test yielded ρ = 0.19 and *p* < 0.02 (under the null hypothesis that ρ = 0) for portraits, and ρ = 0.55 and *p* < 0.003 for quickly snapped photographs. On the other hand, frontal posed photographs did not show a significant correlation.

We additionally looked at the population of artists to determine if they showed individuality and choice in terms of these variables. An illustration of the individuality encountered in our data is shown in Figure 4D. This figure shows data for three example artists, namely, Antonello da Messina, Hans Memling, and Andrea del Castagno. The Imbalance and Asymmetry Indices for these artists appeared to cluster in different, small sub-regions of the scatterplot in the figure. For example, the da Messina portraits appeared to be less asymmetric than the others were. In turn, those by del Castagno appeared to have less imbalance. To confirm these appearances, we used two-sided Mann-Whitney tests to compare the indices of Imbalance and Asymmetry for these three painters. We found that there was no significant difference in imbalance between Messina and Memling. On the other hand, Castagno had lesser imbalance than both Messina and Memling (*p* < 0.007 and *p* < 0.001 respectively). In terms of asymmetry, da Messina and del Castagno did not show a difference. However, both da Messina and del Castagno had less asymmetry than Memling did (*p* < 0.003 and *p* < 0.02 respectively). This sample of artists illustrates that master painters show a complex relationship in terms of their asymmetry and imbalance. The paintings do not necessary show overlap in statistical properties, indicating individual choices for each artist.

**Figure 5:**
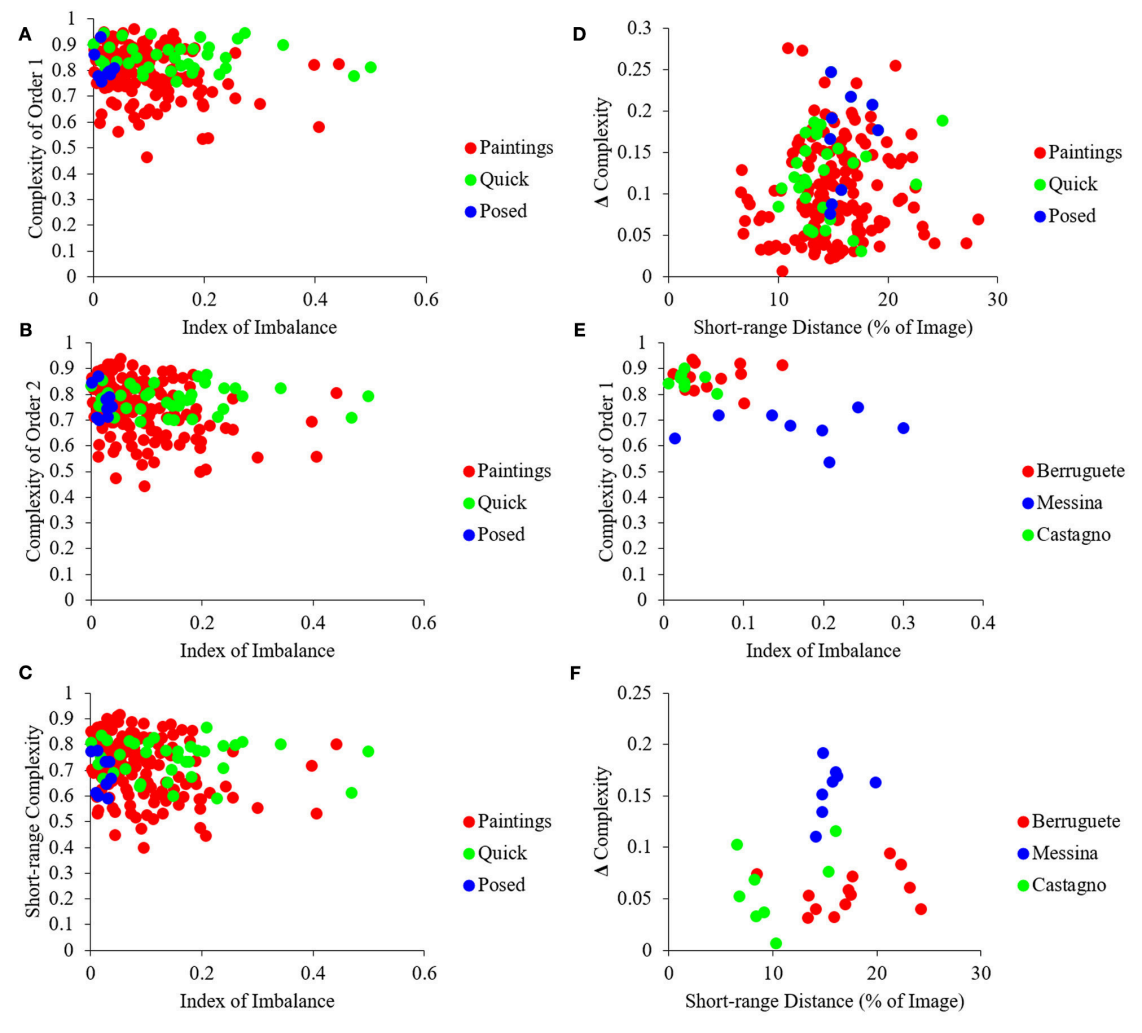
**Quantification of different measures of complexity (see Figure 1 for definitions)**. **(A)** Same as Figure 4A but for Complexity of Order 1 vs. the Index of Imbalance. **(B)** Same as **(A)** but for Median Complexity of Order 2. **(C)** Same as **(A)** but for Short-range Complexity. **(D)** Same as **(A)** but for Δ Complexity vs. Short-range Distance. **(E)** Same as **(A)** but for three example artists, namely, Pedro Berruguete, Antonello da Messina, and Andrea del Castagno. **(F)**, Same as **(D)** but for these three artists. Although many paintings have lower complexity in terms of intensities than all photographs **(A)**, after spatial organization, many paintings become more complex than the photographs **(B,C)**. As in Figure 4, artists “reside” in different areas of the possible values, indicating individuality and choice **(D,F)**. (See text for more details).

Next, we looked at the different measures of complexity to test whether master painters showed biases to increasing it, tried to optimize it, or made individual choices about it. In addition, we investigated the relationship between different indicators of complexity and the Index of Imbalance (Equation 4). One of the indices of complexity that we studied was that of Order 1, which captured the richness of the distribution of intensities (Equation 11). We also studied Complexity of Order 2 (Equation 12). This index captured how much spatial organization reduced the amount of information in the painting. As Equation 12 indicates, Complexity of Order 2 is a function of the Transformation T. Because an infinite number of such transformations are possible, Complexity of Order 2 is not just a simple index (Figures 1E,F). We thus defined in Figures 1E,F several indicators that captured the dependence of Complexity of Order 2 on the transformation. One of those indicators was the median of Complexities of Order 2 across all possible transformations. Another index was the Short-range Distance, i.e., the range of distances for which Complexity of Order 2 varied with displacement. Next, we defined Short-range Complexity, i.e., the median Complexity of Order 2 within the Short-range Distance. Finally, we defined “Δ Complexity” as the difference between Complexity of Order 1 and Short-range Complexity. The results of the analysis using these various indicators of complexity can be found in Figure 5.

The results in Figure 5 showed that artists composed their portraits such as to increase their complexity. To understand this conclusion, begin by considering Complexity of Order 1 (Figure 5A). This complexity showed substantial overlap in portrait paintings, quickly snapped photographs, and frontally posed controls. However, a substantial number of paintings had less Complexity of Order 1 than the other classes of images (Figure 5A). Statistically, portrait paintings had less Complexity of Order 1 than quickly snapped photographs (two-sided Mann-Whitney test, *p* < 0.004). We address a possible reason for this “deficiency” of portrait paintings in the next section. Here, we simply point out that the deficiency disappears when one takes the spatial organization of the painting into account. Complexity of Order 1 refers to the distribution of intensities regardless their spatial organization (Figures 1A–D). In turn, Complexity of Order 2 also takes into account how much spatial organization simplifies the image. (Thus, Complexity of Order 2 is always smaller or equal than Complexity of Order 1—Equation 13.) As observed in Figures 5B–D the Complexity of Order 2 of paintings is no longer less than that of quickly snapped photographs and frontally posed controls. The disappearance of the complexity deficit of paintings by spatial composition was confirmed statistically. Not only were paintings not lesser in complexity, but as seen in Figures 5B–D many of the portraits had more complexity than all quickly snapped photographs and posed controls. Consequently, artists appeared to be making special efforts to maintain a high degree of complexity through spatial composition.

Unlike the Index of Asymmetry that correlated with the Index of Imbalance (Figure 4C), complexity did not exhibit the same dependence (Figures 5A–C). This lack of correlation showed that different fluency variables related to each other in distinct manners. The complicated interdependence between the fluency variables extended itself further. For example, when we made a scatterplot of Δ Complexity vs. Short-range Distance for portrait paintings, we observed a “triangular” shape (Figure 5D). This “triangle” had a broad distribution of Short-range Distances for low Δ Complexity and narrow distribution for large Δ Complexity. This “triangle” shows that the values attained by fluency variables not only have complicated interdependence but also form a space with intricate geometry.

Finally, we investigated whether master painters made individual choices about complexity, as they did for asymmetry and imbalance (Figure 4D). The results in Figures 5E,F show that complexity is also part of the options from which individual artists choose. These figures show data for three example artists, namely, Pedro Berruguete, Antonello da Messina, and Andrea del Castagno. As for asymmetry and imbalance, the complexity indices for these artists appeared to cluster in different, small sub-regions of the scatterplots in the figure. For example, da Messina portraits have less Complexity of Order 1 than those of his counterparts (Figure 5E; two-sided Mann-Whitney tests, *p* < 0.0002 for Berruguete and *p* < 0.0002 for del Castagno). Hence, da Messina made paintings with less range of intensities. Moreover, da Messina had larger Δ Complexities that Berruguete and del Castagno (Figure 5F; two-sided Mann-Whitney tests, *p* < 0.0002 for Berruguete and *p* < 0.0004 for del Castagno). Thus, da Messina not only used fewer intensity values but also did not use spatial composition to compensate and maintain complexity as high as possible. In turn, del Castagno painted his portraits with smaller Short-range Distances than Berruguete (Figure 5F; two-sided Mann-Whitney test, *p* < 0.004). This meant that the latter did not choose to organize his paintings with as small features relative to the canvas as del Castagno did.

Further evidence of individuality with respect to complexity came from an analysis of the correlation between Complexity of Order 1 and Δ Complexity. This analysis revealed a statistically significant negative correlation of −0.47, (*p* < 2 × 10^−09^ – Pearson linear correlation coefficient). Therefore, artists who painted their works with a greater range of intensities tended to have greater spatial complexity in their paintings as well. Interestingly, there were no significant correlations for Complexity of Order 1 and Δ Complexity in posed controls and quickly snapped photographs. This finding provides further evidence for individuality on the part of artists to manipulate the levels of complexity in their works.

### Effects of pose and painting medium

We have seen that when separated by artist, the distribution of the values of the fluency variables is not homogenous (Figures 4D–F). This inhomogeneity indicates that artists, while limited to the boundaries of the overall spread of values, can reside in specific regions of it. Therefore, there is a case for artistic individuality. This individuality may be attributed to a multitude of factors. They include among others the artist's perceptual system, their own preferences, and external influences and norms (see Discussion). We wanted to explore what might cause artists to exhibit their individuality in portrait paintings instead of simply maximizing fluency. While there could be many different mechanisms to their individuality, we looked mainly at two, namely, the interdependence of fluency variables and external constraints to the painting. Here, we explain the interdependence issue through the orientation of pose in portraits. Later, we explain the effect of external constraints through the choice of painting medium.

**Figure 6:**
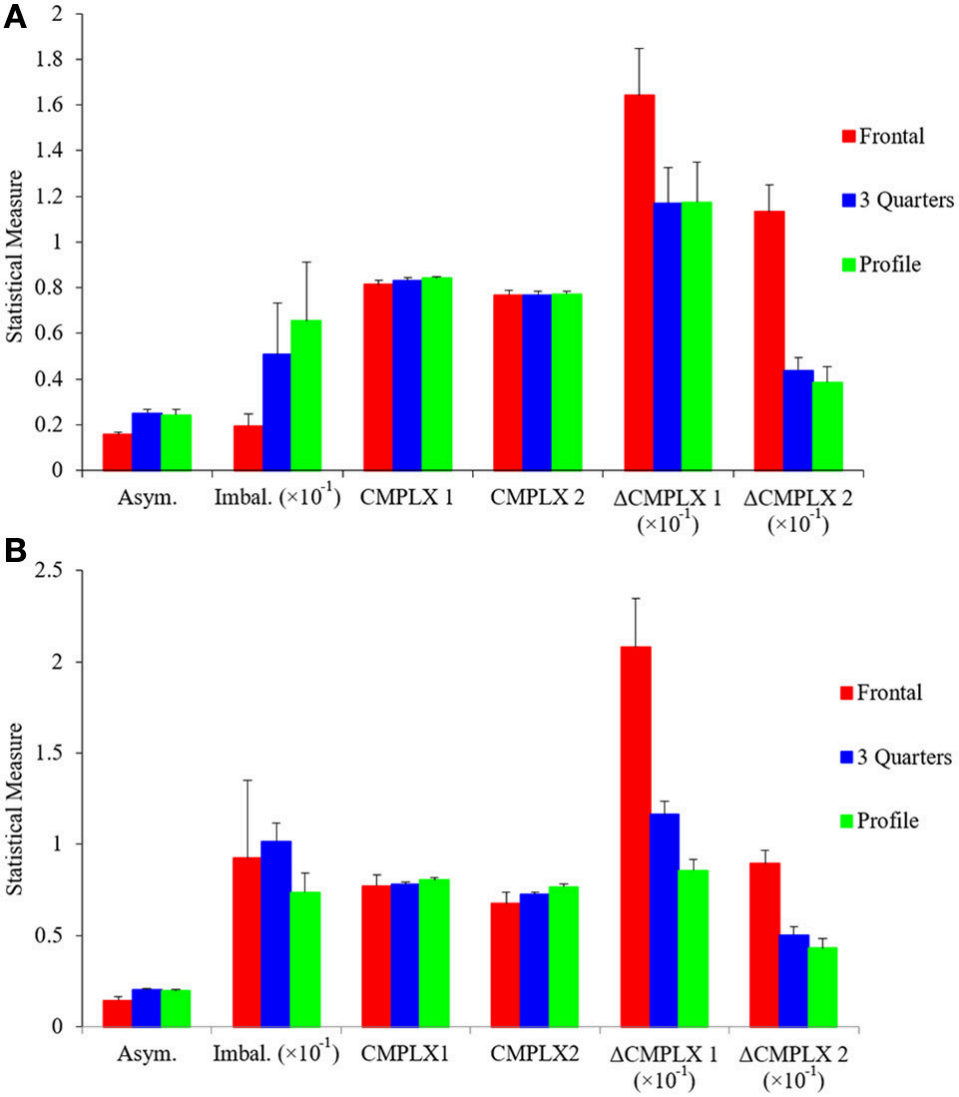
**Different measures of asymmetry, imbalance, and complexity as a function of pose**. **(A)** Control photographs and **(B)** portrait paintings at frontal (0°), 3 quarters (45°), and profile (90°) poses. Horizontal-axis categories are Asym. (Index of Asymmetry—Equation 1), Imbal. (Index of Imbalance—Equation 4), CMPLX1 (Complexity of Order 1—Equation 11), CMPLX2 (Median Complexity of Order 2—defined in Figure 1 from Equation 12), ΔCMPLX1 (Δ Complexity—defined in Figure 1 from Equation 12), and ΔCMPLX2 (same as Δ Complexity but starting from the Median Complexity of Order 2 instead of Complexity of Order 1). These statistical measures are shown as means and standard errors. Results were largely similar for both control photographs and for portrait paintings. Symmetry was lowest in frontal poses, with no difference between 45° and profile poses. Complexity of Order 1 and Median Complexity of Order 2 were similar for all three poses. Higher Δ-complexity values meant a greater loss in complexity, thus frontal poses were the least complex. The only difference between control photographs and portrait paintings was in respect to balance. There was lower balance in the frontal poses in the former but not the latter.

If two fluency variables are interdependent, they may force the artist to choose, because increasing fluency through one variable may reduce fluency through another. We saw an example of interdependence in the positive correlation between symmetry and balance (Figure 4C). However, that correlation was positive, thus increasing fluency through symmetry helped increase fluency through balance. To find an example of a negative correlation, we resorted to pose. As suggested by Figure 1, frontal poses appear to reduce complexity more than other poses, because the former tend to be symmetric in portraits. In symmetry, a pixel predicts another after a reflection transformation. This extra predictive power explains the lowering of Short-range Complexity for the frontal but not the profile painting in Figure 1 (compare the blue and green lines in Figures 1E,F). To quantify this apparent effect, we looked at how the different measures of asymmetry, imbalance, and complexity vary with controlled photographs taken at frontal (0°), 3 quarters (45°), and profile (90°) poses. The results can be seen in Figure 6.

Figure 6A shows that the Indices of Asymmetry and Imbalance are negatively correlated with Δ-complexity measures when poses are changed, forcing artists to choose between them. Frontal poses were the least asymmetric (one-sided one-way ANOVA, 26 degrees of freedom, *F* = 7.41, *p* < 0.002), followed by 3 quarters and profile. (Intriguingly, none of our results showed any differences between the 3 quarters or profile poses.) Similarly, frontal poses were significantly less imbalanced than the other two (one-sided one-way ANOVA, 26 degrees of freedom, *F* = 3.14, *p* < 0.03). For Complexity of Order 1 and Median Complexity of Order 2, the values were nearly identical across the poses. This similarity was expected for Complexity of Order 1, because the distribution of intensities did not change drastically with pose. However, the Median Complexity of Order 2 result was more unexpected. It probably reflected large spatial features in the canvases, as the complexity lines plateau for large displacements (Figures 1E,F). In contrast, the Δ-complexity measures gave, by definition, a more sensitive measure of complexity at finer spatial scales. Our results showed that frontal poses yielded larger Δ-complexity measures than the other two (e.g., one-sided one-way ANOVA, 26 degrees of freedom, *F* = 23.5, *p* < 2 × 10^−6^ for ΔCMPLX2). Thus, frontal poses were the least complex at short-range scales.

We observed similar results when subjectively classifying poses in portrait paintings (Figure 6B). Frontal poses were statistically less asymmetric and yielded larger Δ-complexity measures than angled and profile poses. Consequently, as for controlled photographs, symmetry was negatively correlated with complexity in portrait paintings as poses changed. However, balance did not show the same correlation. This was likely due to portrait paintings not being exactly frontal like our control images. Our balance measure was sensitive to artistic choices such as very slight head tilts observed in these paintings.

**Figure 7:**
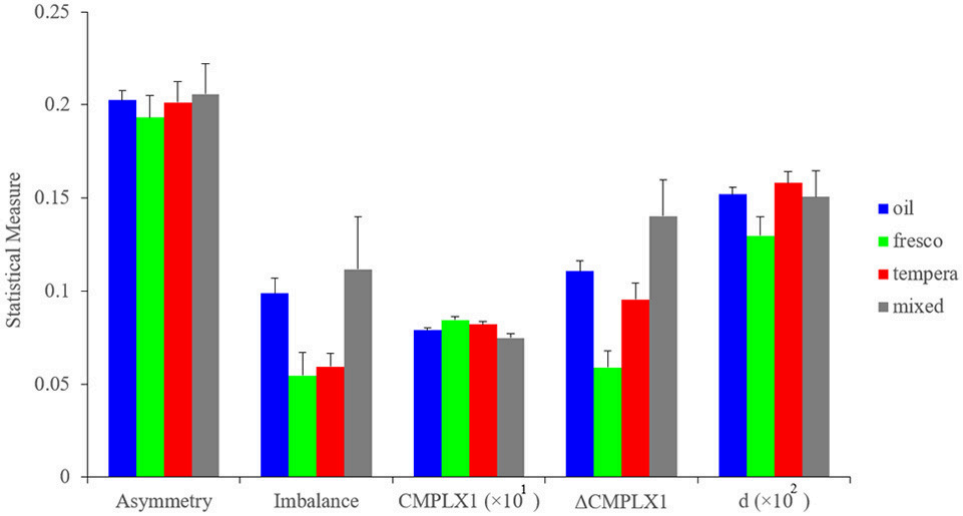
**Measures of asymmetry, imbalance, and complexity as a function of the painting medium**. Horizontal-axis categories are as in Figure 6, except for d, which is Short-range Distance presented as the percentage of the size of the canvas (defined in Figure 1 from Equation 12). These statistical measures are shown as means and standard errors. The measures show that during the Early Renaissance, painting media affected some fluency variables (e.g., balance, Complexity of Order 1, Δ Complexity, and Short-range Distance). However, the painting media did not affect all variables (symmetry).

External factors, such as the painting medium that artists must use also force them to make choices. During the Early Renaissance, artists mainly had a choice between a handful of media (Bambach, 1999). If a patron wanted the artist to paint a wall of a church, for example, then the painter had to use fresco techniques. These allowed the painting of small features relative to the large image but were less flexible than oil or tempera. In contrast, these latter techniques but not fresco allowed retouching of the canvas, giving the artist the ability to experiment. However, in Early Renaissance, some pigments for oil were expensive or difficult to get. Hence, the artist had to take into account costs and richness of materials to choose how to make his paintings. Our database contained paintings in fresco, tempera, oil, or mixes of oil and tempera. We investigated how each of the fluency variables differed in these painting media. The results can be seen in Figure 7.

Our analysis showed that the artists' works were influenced by the painting medium. For example, both oil and mixed paintings were statistically significantly more imbalanced than frescos and tempera paintings (two-sided Kruskal-Wallis test, 153 degrees of freedom, χ^2^ = 11.9, *p* < 0.008). In turn, fresco paintings had greater Complexity of Order 1 (two-sided Kruskal-Wallis test, 160 degrees of freedom, χ^2^ = 9.86, *p* < 0.02) and the least Δ Complexity (two-sided Kruskal-Wallis test, 153 degrees of freedom, χ^2^ = 19.8, *p* < 0.0002). These results indicated that frescos yielded paintings that were more complex than the other media. In contrast, there was no significant difference in asymmetry across media. The Discussion will analyze the some of the possible reasons for how different media affect different fluency variables in distinct ways.

## Discussion

### Expanding the PFT: the neuroesthetic-space principle

In this study, we examined whether master portrait painters from the Early Renaissance Period showed esthetic biases according to the PFT. The theory posited that increased perceptual fluency was associated with a greater esthetic response. Therefore, one would expect that the painters created their works in a way to maximize the fluency of perceptual variables. In particular, painters might try to optimize balance, symmetry, and complexity in their works. These biases might be the result of conscious intent or occur unconsciously. Overall, we found that painters were indeed displaying esthetic biases toward increasing fluency. The painters' work exhibited more symmetry and balance than quickly snapped, non-artistic controls (Figures 3, 4). Moreover, the results indicated a deliberate effort to increase the complexity of paintings through careful compositions (compare Figures 5A–D). However, we also found that the painters were not optimizing all of their fluency variables independently of each other, as shown by our comparison to posed control photographs (Figures 3, 4). Why was the optimization not applicable to all fluency variables?

To explain why master painters may not be optimizing all of their fluency variables independently, we propose to extend the PFT with the Neuroesthetic-Space Principle. This principle begins by proposing that during an esthetic judgment, the brain decomposes an image into its fluency variables. Our equations give possible definitions for some of these variables, but their exact details depend on the neural mechanisms performing the computations. In this work, we explore three such variables, namely, balance, symmetry, and complexity, but the Space is likely much more complex, being highly multi-dimensional. Our results suggest that the possible values that these variables may attain are not likely boundless. Instead, they may reside in a certain limited space. Cross sections of this space are illustrated by the scatterplots in Figures 4A, 5A–D. We term this the Neuroesthetic Space. Therefore, the Neuroesthetic Space defines the range of possible values of all perceptual variables involved in mediating the fluency of a sensory input (an image in our case). Our data show that different painters reside in different sub-regions of this Space (Figures 4B, 5E,F). We propose that the individuality of painters within the Neuroesthetic Space is a major reason for why they do not necessarily optimize each fluency variable.

### Artistic individuality within the neuroesthetic space

Why did master painters exhibit individuality within the Neuroesthetic Space? We already mentioned that painters could use different painting media. However, the choice of these media was sometimes imposed by the artists' patrons and sometimes dependent on accessibility. For example, in the Early Renaissance, some artists had to do frescos, while others could use oil or tempera (Paoletti and Radke, 2005). We found that the painting media had an effect on the artistic output. Across all paintings and artists, our results indicated that oil-based and mixed paintings had the greatest amount of imbalance (Figure 7). In contrast, frescos showed the greatest amount of complexity (Figure 7). The latter effect had a simple explanation: using a large fresco as a medium, painters could increase the amount of detail and relative finer features otherwise not possible with alternate media. However, we have no explanation yet for the greater imbalance in oil-based paintings. Artists used oil, since both oil and tempera allowed retouching, thus affording greater freedom in the composition of paintings (Bambach, 1999). But oil painting was new in the Early Renaissance and thus getting certain types of pigments was expensive and difficult (Kirby, 2000). This imposed a limitation in the complexity of oil paintings, likely preventing this variable from reaching optimal values (Figure 7). Hence, painting media could have a measurable impact on the perceptual characteristics of a painting and thus, in which region of the Neuroesthetic Space an artist resided.

Another reason for individuality in the Neuroesthetic Space is that some fluency variables show interdependence. If all the variables were independent, then painters could manipulate them individually; nonetheless, with interdependence, the optimum of one variable might not be the optimum of another. Thus, artists had to choose. An example of a mild interdependence affecting artists' choice was the weak correlation between symmetry and balance (Figure 4). Another stronger correlation in portraiture was forced by pose. Considering that a portrait was a relatively simple art form, with its inherent qualities such as the natural symmetry of the human body and face, artists needed to innovate to increase the amount of information and complexity to appeal to the viewer. One simple way to do so was to change the orientation of the pose. By changing the pose away from frontal, artists effectively increased the level of complexity (Figures 6); however, as a byproduct, they also increased asymmetry and imbalance (Figure 6). Portrait painters in the Early Renaissance generally chose to avoid frontal poses, implicating that in part, complexity was an important factor for them (Pope-Hennessy, 1966). The exact equilibrium between complexity on one hand and symmetry and balance on the other was an individual choice (Figure 1). Artists, by choosing which variables they emphasized more, ended up in different sub-regions of the Neuroesthetic Space.

Therefore, because of media constraints, fluency-variable interdependence, or other reasons, portrait painters must exert individual choice. The difference in choices undoubtedly has innate, physiological, and personal experience components. For example, studies of preference for complexity show that different people lie in distinct portions of an inverted “U” curve (Jacobsen and Höfel, 2002; Güçlütürk et al., 2016). Thus, some individuals enjoy moderate increases in complexity from a starting point, while others dislike the same changes. This difference in enjoyment of complexity can be attributed to various factors including sex, age, personality and even physiological differences in visual perception (Crosson and Robertson-Tchabo, 1983; Chamorro-Premuzic et al., 2010; Vaughn et al., 2013; Spehar et al., 2015). However, the difference can also be due to experience and exposure, as when comparing artists and non-experts (Smith and Melara, 1990). The thresholds for asymmetry and imbalance may be lower in painters, and their desire for complexity higher (McWhinnie, 1968; Pang et al., 2013; Else et al., 2015). Furthermore, numerous neurological studies have shown how alterations in the perceptual system of painters can lead to an entirely different type of art (Chatterjee, 2004a).

### Determination of the fluency variables of the neuroesthetic space

An innovation of our paper is the introduction of computational methods to begin outlining the relationship between multiple fluency variables in a Neuroesthetic Space. Other definitions of the same fluency variables are possible and exist, of course (Dakin and Watt, 1994; Wilson and Chatterjee, 2005; Forsythe et al., 2008). However, we feel that our definitions are on the right track. This feeling stems from two reasons: First, the sub-regions occupied by individual artists within the hypothesized Neuroesthetic Space are small (Figures 4D, 5E,F). Therefore, there is reason to believe that our measures capture visual properties that are important to the artists and the observers. Nevertheless, this assertion rests on the assumption that our measures are perceptually realizable to both artists and observers alike. Future studies could explore this notion of “esthetic sensitivity” to our specific variables with psychophysical measures and tests (Götz, 1985; Wilson and Chatterjee, 2005; Chatterjee et al., 2010; Samuel and Kerzel, 2013). Second, the definitions of our variables are based on knowledge from basic neuroscience. They arise from previous studies that have specifically compared objective cognitive-neuroscience measurements to subjective esthetic ratings. For example, we can relate our measure of symmetry to one of the earliest definitions in the field of esthetics (Mach, 1914; Birkhoff, 1933). Additionally, studies investigating visual perception, esthetic preferences, and neural correlates of beauty have all used similar notions of reflectional bi-lateral symmetry (Mach, 1914; Wenderoth, 1994; Jacobsen and Höfel, 2002; Jacobsen et al., 2006; Makin et al., 2012; Mayer and Landwehr, 2014).

As for refining the definition of the fluency variables, a process such as the one that we used for balance suggests a path to follow. Balance is widely attributed as necessary to the appreciation of an image. Experiments have shown that lack of balance can be detected in the order of just tens of milliseconds (Locher and Nagy, 1996). While the colloquial definition of balance is generally agreed upon, there remains a lack of a comprehensive objective measurement for balance (Hübner and Fillinger, 2016). To date, the most influential theory of visual balance was derived from Arnheim, who posited a physicalist-like definition (Arnheim, 1954). In our study, we sought to test his theory and two other measures of balance, the Integral and Mean methods. The integral method computes balance through summing the relevant property (here, intensities) on each side of the balance line. In turn, the mean method compares the mean of the property on the two sides. To test these three methods, we investigated at which point in the image each method led to perfect balance, i.e., the position of the balance lines. We then predicted that the best balance lines should fall near the most important parts of the portrait. Thus, these lines should be near the key facial features of the subject (Walker-Smith et al., 1977). Previous eye-tracking studies have shown that humans tend to look primarily at these features (e.g., the eyes) when observing portraits (Massaro et al., 2012). As Figure 1 illustrates, of all of the balance lines, the integral line was the closest to the main features of the face. This result indicated that the integral line was the closest to what artists may use unconsciously. Thus, we had our first indication that the balance line was the correct one to use throughout the paper.

To test further whether the integral definition of balance was meaningful, we measured the quality, i.e., the precision, of the ensuing balance lines. The results of a row-by-row analysis in Figure 2 showed that master painters had a bias toward balance everywhere in the canvas. This precise and thorough balancing suggested that integral balance approximated the process in the minds of artists. Therefore, we used the integral definition to evaluate the balance of different paintings.

However, although we gained confidence about the integral balance, we know that our definition is probably not as good as it could be. If it were, we would expect the bar plots in Figure 2 to show a larger advantage for the correct definition of balance over the physicalist and the mean ones. Some simple tweaks to the model could help improve the balance model. For instance, all of our methods are based on the assumption that the balance of an image is reliant primarily on its intensity values. But the calculation of saliency underlying the computation of balance uses other visual properties besides intensities (Itti et al., 1998). These properties include, e.g., color contrasts and orientation edges. Perhaps enriching the set of visual properties used in our model of balance could help improve the results significantly.

Finally, how confident can we be of our definitions of complexity? The debate regarding a good psychophysical definition of visual complexity has remained unresolved thus far. Most existing definitions are grounded in some manner in information theory (Shannon and Weaver, 1949). These include looking at the amount of objects and elements in the image, their regularities, methods of image compression, and exploring amplitude spectra and fractal scaling (Donderi, 2006; Forsythe et al., 2011). These definitions have been extensively used in studies investigating subjective measures of interest, beauty, art production, and detection thresholds (Vitz, 1966; Nadal et al., 2010; Güçlütürk et al., 2016). For example, artistic paintings, including portraits have similar power spectra as natural images (Graham and Field, 2007; Redies et al., 2007b). Both artistic and natural spectra tend to fall as the square of the spatial frequency. Such a tendency indicates the fractal property of self-similarity. That both art and natural images exhibit self-similarity is interesting in the context of our findings. We show a different behavior in artistic and natural portraits (Figures 5A–D). The reason for this difference is that power spectra and our definitions of complexity capture different aspects of images. The power spectrum is the Fourier transform of the autocorrelation function. Hence, power spectra capture statistics of the multiplication of intensities in *pairs* of pixels when *translating* an image relative to itself. The pairwise multiplication means that power spectra do not capture first-order statistics as our Complexity of Order 1 does. Similarly, the relationship to autocorrelation means that power spectra only capture translational statistics, as opposed to the arbitrary isometric transformations measured by our Complexity of Order 2. Hence, for instance, Complexity of Order 2 captures transformations such as reflection, thus being sensitive to the effect of symmetry on complexity (Figure 1). Moreover, that our measures of complexity involve the logarithmic non-linearity inherent in information theory (Equations 10–12), they can capture statistics higher than those of second order.

### Human neuroscience of the PFT and the neuroesthetic space

The focus of our study is primarily regarding the exteroceptive variables of perception, termed “outer psychophysics” by Fechner (Fechner, 1965). While we have not experimentally addressed the “inner psychophysics” or neural mechanisms, we discuss them next.

What are the possible neural mechanisms of the PFT and the Neuroesthetic Space? We begin with the latter. Models of esthetics usually account for dual levels of processing, e.g., a “lower” sensory and a “higher” cognitive route. The latter which includes semantic and emotional content (Chatterjee, 2004b; Leder et al., 2004; Locher et al., 2007; Leder, 2013; Chatterjee and Vartanian, 2014; Redies, 2015). In turn, the Neuroesthetic Space highlights the importance of the former, the perceptual stream of esthetic processing. This space represents the decomposition of the sensory input, which is the image in our case, into its fluency variables. Therefore, the Neuroesthetic Space should have a distributed representation across the brain. The view that esthetic judgments occur through a hierarchy and over a distributed network has widespread support (see above). For instance, the symmetry component of the Neuroesthetic Space is processed in several areas of the extrastriate visual cortex. They include specially V3A, V4, V7, and LO, as well as visuospatial regions, such as superior parietal lobule and intraparietal sulcus (Jacobsen et al., 2006). In turn, the computation of saliency, i.e., the detection of imbalance requires interplay between the visual cortex and the lateral geniculate nucleus of the thalamus (O'Connor et al., 2002). Finally, the fluidity of complexity, i.e., the ease of flow of information is part of the design of multiple parts of the visual system. Some of its circuitry is specially designed to maximize the flow of information given limitations of the neural hardware (Atick and Redlich, 1992; Balboa and Grzywacz, 2000).

We now turn our attention to the possible mechanisms of the PFT. To understand them, we reiterate that the computation of the location of the sensory input within the Neuroesthetic Space is distributed. Consequently, multisensory integration is required for the estimation of overall fluency and thus, esthetic processing. While the idea that an esthetic judgment involves distributed computations and subsequent integration is largely accepted, its mechanisms are unclear (Redies, 2015). Because art activates the brain across multiple modalities and networks, studying the underlying processing streams independently has been difficult. The consensus, however, is that the perceptual network must somehow interact and integrate with the cognitive and affective networks. This interaction is supported by experimental evidence (Kontson et al., 2015). While many models of the interaction between these two domains exist and their details overlap, the model proposed by Brown et al. used a meta-analysis of neuroesthetic studies as its basis and is thus especially interesting (Brown et al., 2011). This model postulates that an esthetic judgment is reached through an interaction of exteroceptive and interoceptive information, mediated by the orbitofrontal cortex, OFC and Anterior Insula respectively. The authors suggest that the OFC is an ideal candidate for the computing site of exteroceptive or perceptual processing. The suggestion stems from the OFC being implicated in both attaching reward to stimuli through appraisal and tracking previous rewards (O'Doherty et al., 2001; Kringelbach and Rolls, 2004). We, therefore, propose that the Neuroesthetic Space is the site of different variables that constitute this exteroceptive input to the OFC. In an esthetic judgment, therefore, an observer first computes each of the variables in this space. The observer then determines a relationship between them that maximizes the reward value in the OFC. This OFC information can then interact with an internal homeostatic state mediated by the anterior insula to result in a specific esthetic response (Brown et al., 2011).

### Computation and dynamics of the neuroesthetic space

In this study, we propose the principle of a Neuroesthetic Space. This space is composed of complex, interdependent perceptual variables. Furthermore, the shape of the boundaries of the space may have complex geometries (Figures 4, 5). Consequently, we may need to build a computational model to understand the optimization of fluency variables in this space. Such a model would help us account for the relationship between these variables. Thus, the model might make sense of the way in which an artist optimizes them jointly (not individually) in the production of art. In particular, we might be able to use the model to gain further insights into how different artists modulate the properties of their work. However, building this model will not be easy, because to make it work for each artist, we will need free parameters related to individuality.

Another question that we face about the principle of Neuroesthetic Space pertains to its temporal dynamics. How do painters and their paintings evolve across art periods? Art could move inside a fixed Neuroesthetic Space, or its boundaries could drift slowly across time or jump abruptly from period to period. We began our analysis focusing on the period when portraiture first emerged. This focus allowed uncovering fundamental fluency variables. By tracking their dynamics over time, we may effectively measure how this art forms and its Neuroesthetic Space changes from period to period (Spehr et al., 2009; Wallraven et al., 2009). Additional such studies could investigate other types of art throughout history and different cultures. Therefore, such studies could give us insights on the universal properties of esthetics and whether our brains differ in time and region in how we create, process, and appreciate art.

## Ethics statement

This study was carried out in accordance with the recommendations of Human Subjects Guidelines, Georgetown Institutional Review Board with written informed consent from all subjects. All subjects gave written informed consent in accordance with the Declaration of Helsinki. The protocol was approved by the Institutional Review Board at Georgetown University.

## Author contributions

HA was involved in the design of the project, developing and using programming scripts for data gathering and analysis, carrying out the measurements with participants, interpreting results, and writing and revising the manuscript. IC-H was involved in the design of the project, selecting the paintings, collecting data, carrying out the measurements with participants, and revising the manuscript. NG was involved in the design of the project, developing the mathematical equations, creating and using programming scripts for data gathering and analysis, interpreting the results, and revising and approving the manuscript for publication.

## Funding

This work was partially funded by the Training Grant T32NS041231 from the National Institutes of Health to the Interdisciplinary Program in Neuroscience at Georgetown University.

### Conflict of interest statement

The authors declare that the research was conducted in the absence of any commercial or financial relationships that could be construed as a potential conflict of interest.

## Footnotes

^1^The attribution of this painting has been in dispute by connoisseurs like Morelli, Springer, and Müntz (Pagden, 1986). It was previously thought to be by either Hans Holbein or Pietro Perugino. More recently, scholars began attributing it to Raphael (Raffaello Sanzio da Urbino) (Dussler and Cruft, 1971). Raphael was an apprentice at the workshop of Perugino and this painting is from that period (Vasari, 2007). Artstor, the source of paintings for this study attributes the portrait to both Perugino and Raphael. We decided to include ie period, and was either by Perugino or under his supervision.
